# Case report: takotsubo syndrome in infectious endocarditis

**DOI:** 10.1007/s00392-020-01629-6

**Published:** 2020-03-31

**Authors:** Manuel Rattka, Jule Gundlach, Wolfgang Rottbauer, Mirjam Keßler

**Affiliations:** Klinik für Innere Medizin II, Universitätsklinikum Ulm, Albert-Einstein Allee 23, 89081 Ulm, Germany

Sirs:

We report a 60-year-old woman with a history of aortic valve replacement (23 mm bio-prosthesis), who was admitted to our emergency unit and suffered from progressive shortness of breath and chest discomfort for about 7 days. The physical examination and the resting electrocardiogram (ECG), which showed a known left bundle branch block (LBBB, Online Resource 1A), were inconclusive. Laboratory tests revealed an increased high-sensitive cardiac troponin T of 76 ng/l (normal < 14 ng/l) and an elevated C-reactive protein of 16.1 mg/l (normal < 5 mg/l). The transthoracic echocardiographic examination (TTE) showed new left ventricular apical dysfunction. Suspecting cardiac ischemia, a cardiac catheter examination, was conducted. The examination confirmed apical ballooning (Fig. 1a, b) without signs of coronary artery disease, leading to diagnosis of takotsubo syndrome (TTS) and hospitalization of the patient. On the third day of hospitalization, the patient developed fever, but further search for the focus of infection (X-ray of the chest, urine test, hemocultures) remained inconclusive. At day 5, she experienced transitory right-sided hemiplegia and global aphasia. Cranial magnetic resonance tomography revealed left-sided embolism of the basal ganglia and the temporal lobe (Fig. 1c). Moreover, an abdominal computer-assisted tomography scan revealed infarction of the spleen and both kidneys (Fig. 1d). Transesophageal echocardiography (TEE) showed no signs of infective endocarditis (IE). Suspecting severe sepsis of unknown origin with multiple embolisms, and because the “Modified Duke Criteria” for IE were not met, an empiric antibiotic therapy with Piperacillin/Tazobactam, Flucloxacillin, and Fluconazole was initiated after interdisciplinary discussion of the case. Repetition of TTE and TEE examinations did not lead to any new findings. The antibiotic therapy was discontinued after approximately 3 weeks due to inefficacy and to resume microbiological testing. At day 30, the ECG displayed a third grade atrioventricular-block and change from a LBBB pattern to a RBBB pattern, leading to repetition of the TEE examination and diagnosis of prosthetic aortic valve endocarditis with valve ring abscess (Fig. 2, Online Resources 1B and 2). Due to third grade atrioventricular block, a transitory cardiac pacemaker was implanted. An antibiotic regime containing ampicillin, flucloxacillin and gentamicin was initiated in consideration of the current guidelines [1]. Hemocultures became positive for methicillin-resistant staphylococcus aureus which led to the initiation of a targeted therapy with intravenous vancomycin, rifampicin, and gentamicin [1]. In the presence of uncontrolled infection surgical valve replacement was executed at day 34. After successful replacement of the infected valve a dual-chamber pacemaker was implanted at day 37. At day 40, the patient dismissed herself from hospital because of rising mental stress due to the long hospitalization. Afterwards, antibiotic therapy with oral rifampicin and intravenous vancomycin, administered in an outpatient setting, was resumed for five more weeks according to our microbiologists’ advise. TTE and TEE before dismissal and at the next follow-up visit showed resolution of IE and TTS.

**Fig. 1:**
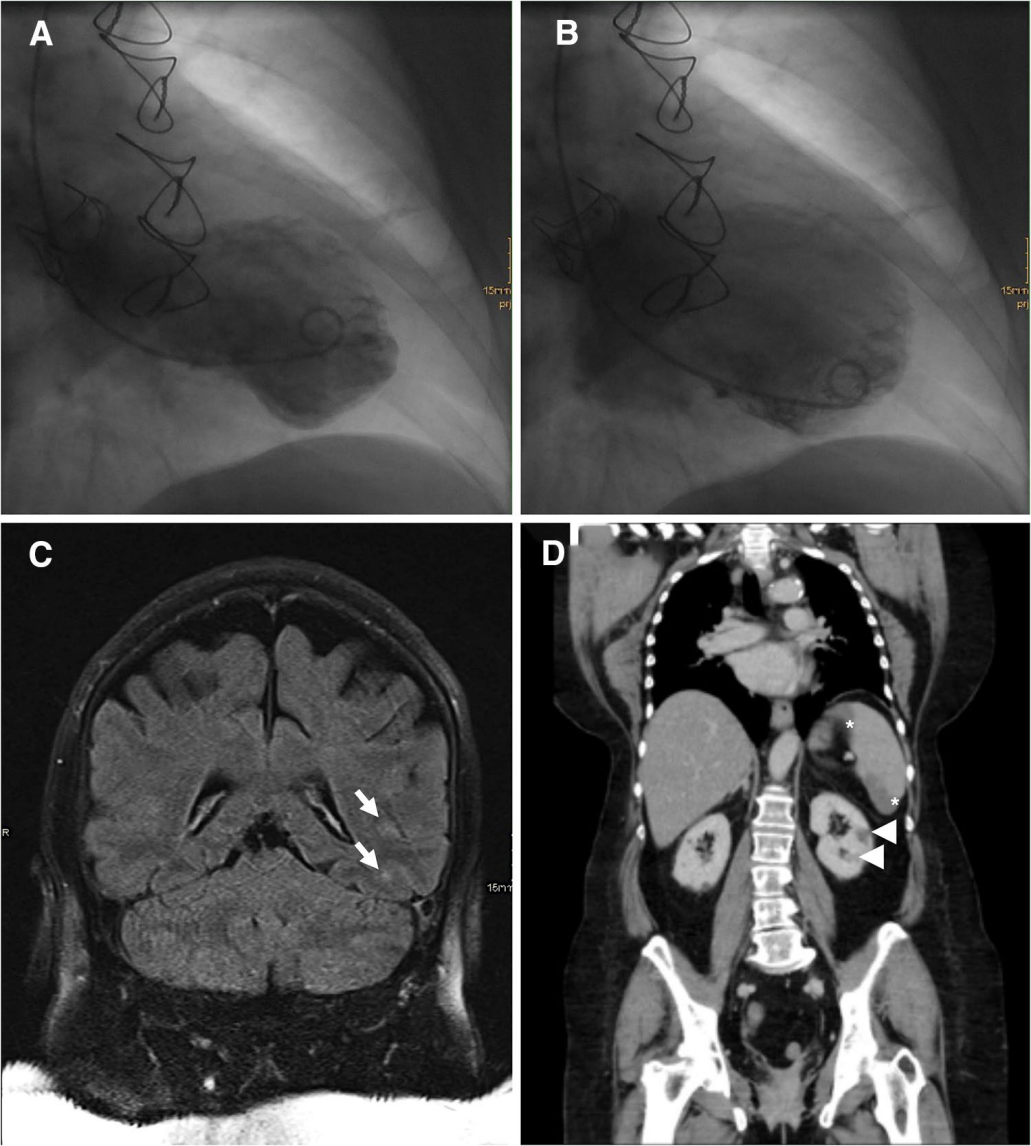
**a** Ventriculography shows end-systolic apical ballooning. **b** End-diastolic ventriculography. **c** Magnetic resonance tomography reveals left-sided temporal embolic ischemic lesions (arrow) and computer-assisted tomography displays splenic (asterisk) and renal (arrowhead) infarction (**d**)

**Fig. 2:**
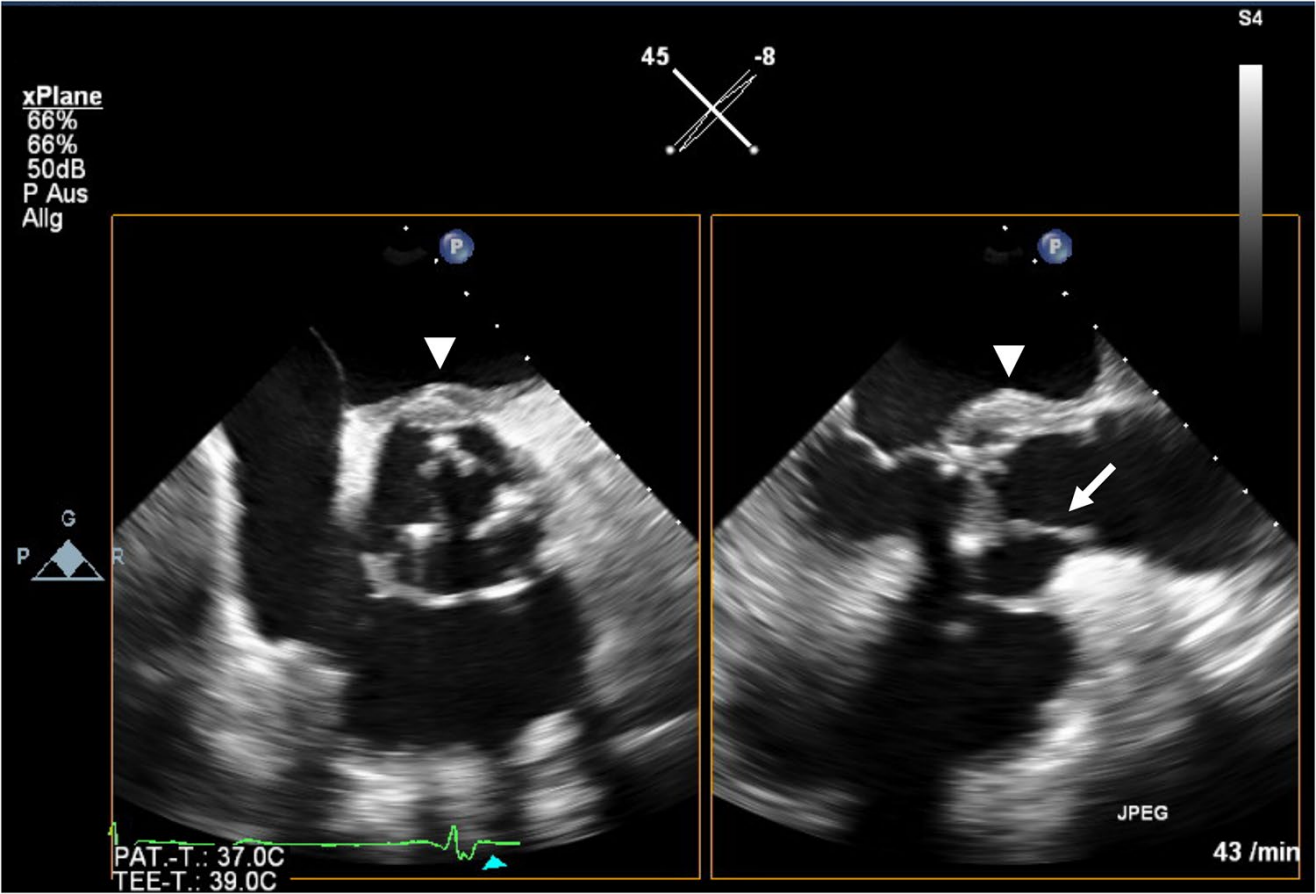
Transesophageal echocardiography shows a hyperechoic mobile mass attached to the aortic valve (arrow) and perivalvular swelling (arrowhead) consistent with infectious endocarditis and concomitant abscess formation

TTS with concomitant IE is a rare phenomenon and the effect of TTS on the course of IE as well as their mechanistic relationship is currently not understood. While it is recognized that emotional and physical stress can lead to TTS the exact pathomechanism is unknown, but recently, the brain–heart interaction is getting more and more into the spotlight [2–5]. Here, we report the first case of TTS in prosthetic aortic valve endocarditis.

TTS usually mimics myocardial infarction involving elevated cardiac biomarkers and ECG changes [6, 7]. In a recent study, the assessment of a diagnostic ECG score could help identifying patients with TTS. As patients with left bundle branch block were not eligible for inclusion, the score could not be applied in the presented case [6, 8, 9]. Moreover, the presence of a LBBB pattern in TTS patients has been associated with a higher incidence of arrhythmia and shock in TTS patients [10]. However, in our patient, the LBBB had already been known since after the first surgical aortic valve replacement and was not related to TTS.

Besides from the presented case of concomitant TTS and IE, only three other cases have been reported, all affecting the mitral valve [11–13]. While in these cases, triggers other than IE such as previous surgical treatment, severe mitral valve regurgitation and meningitis might have contributed to TTS development, in our case, despite a time-to-diagnosis of 30 days, there were no other TTS triggers besides from severe IE with embolic events [11–13]. Previous studies showed that in about 15% of cases of IE, the initial TEE is negative, especially in the presence of pre-existent lesions, such as prosthetic valves [14]. Furthermore, in our case successful surgical and antibiotic therapy for IE resulted in resolution of TTS, thereby substantiating the correlation between IE and TTS.

Septic embolism, a sign of uncontrolled infection, affects the patients’ prognosis and presents a common cause for surgery in IE [1]. In three of the four reported patients, cerebral and abdominal imaging was performed, showing embolic events affecting the brain, spleen and kidney [12, 13]. Hence, self-limiting embolic cardiac infarction, even if unlikely, cannot be ruled out as an explanation for apical left ventricular dysfunction. However, it is more likely that septic embolism and excessive bacteremia increased physical stress, thereby triggering the pathogenic mechanisms of TTS [15]. Nevertheless, there is another plausible and interesting mechanism possibly inducing TTS in IE patients. As previously stated, TTS has been associated with central nervous system disorders involving regions responsible for emotional processing [2–5]. Thus, it is noteworthy to mention that in the patients who received cerebral imaging cerebral embolisms were detected, amongst others affecting the temporal lobe [12, 13]. Therefore, it is conceivable that cerebral embolisms caused by IE might precede TTS development. This supports the theory that the central nervous system, and especially, the parts involved in generation and processing of emotions are of utmost importance for TTS development [2–5].

TTS is potentially life threatening and features comparable in-hospital mortality as myocardial infarction [16]. The prognosis of IE and concomitant TTS is unknown. IE is a state of systemic inflammation due to bacteremia and possibly septic patients with TTS display a suitable cohort to draw comparisons. Recently, a study analyzed the outcome of patients with TTS and sepsis and showed that patients with coexisting TTS were predominantly female and had a higher rate of pre-existing anxiety disorders [15]. Interestingly, the presence of TTS was not associated with a higher in-hospital mortality [15]. Similarly, all four patients with TTS and concomitant IE were female and had a favorable outcome [11–13]. All received antibiotic as well as surgical treatment. In three cases, the affected valve was replaced while in one the native valve was reconstructed [11–13]. As a consequence, TTS in IE patients might not worsen the patients’ prognosis if IE is treated sufficiently.

In conclusion, the unusual case provides three messages. TTS can display an early sign of IE, especially if the patients show signs of systemic inflammation. Contrasting the general statement that heart failure in IE patients has an adverse impact on the prognosis, TTS might not worsen the outcome if IE is treated sufficiently. Possibly, TTS in IE patients can be triggered by septic emboli affecting cerebral areas involved in emotion regulation and should be regarded as a sign of uncontrolled infection, which might influence the treatment strategy.

## Electronic supplementary material

Below is the link to the electronic supplementary material.

Supplementary file1 Online Resource 1: (A) ECG at admission shows a sinus rhythm and a known left bundle branch block. (B) At day 30 of hospitalization the ECG reveals a new third grade atrioventricular block (arrow, p-waves) and a right bundle branch block. (PDF 955 kb)

Supplementary file2 Online Resource 2: Transesophageal echocardiography is consistent with diagnosis of infectious endocarditis and concomitant abscess formation. (MP4 4043 kb)
