# Supplementary figures and images for: Case report: takotsubo syndrome in infectious endocarditis
Source: Clin Res Cardiol. 2020 Mar 31;109(9):1193–6. doi: 10.1007/s00392-020-01629-6 (PMC7449991; doi:10.1007/s00392-020-01629-6)

**A**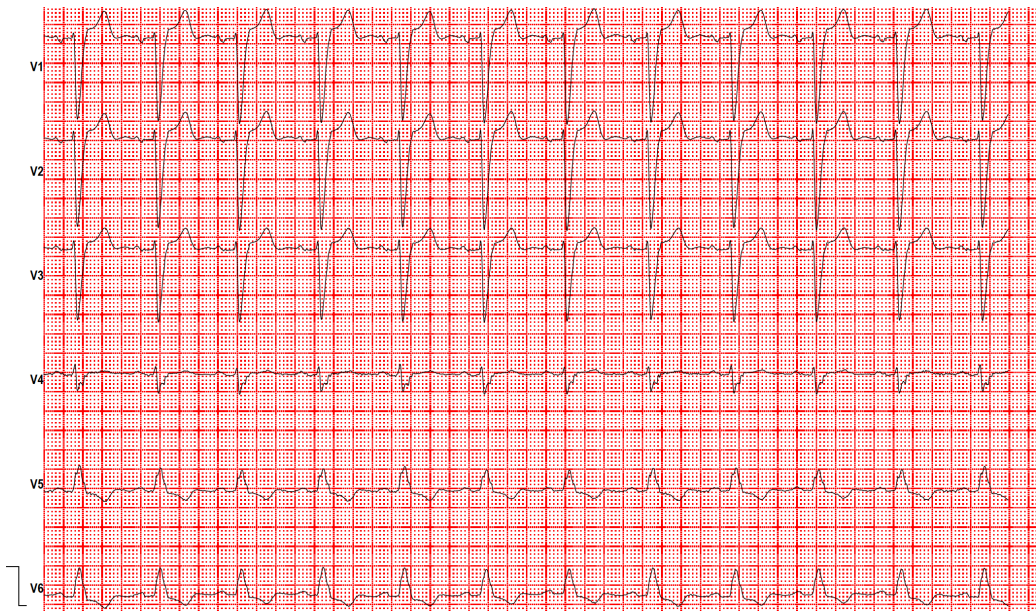**B**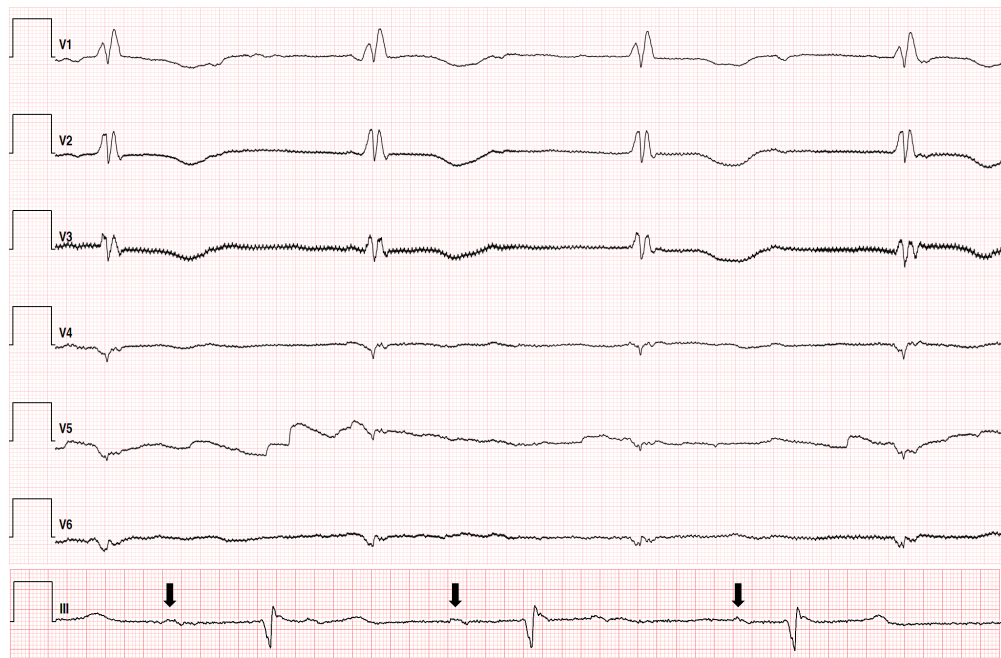

Supplement: Supplementary file 1 — Supplementary file1 Online Resource 1: (A) ECG at admission shows a sinus rhythm and a known left bundle branch block. (B) At day 30 of hospitalization the ECG reveals a new third grade atrioventricular block (arrow, p-waves) and a right bundle branch block. (PDF 955 kb) [file 392_2020_1629_MOESM1_ESM.pdf]
